# Size Matters? Penis Dissatisfaction and Gun Ownership in America

**DOI:** 10.1177/15579883241255830

**Published:** 2024-05-31

**Authors:** Terrence D. Hill, Liwen Zeng, Amy M. Burdette, Benjamin Dowd-Arrow, John P. Bartkowski, Christopher G. Ellison

**Affiliations:** 1 Department of Sociology and Demography, The University of Texas at San Antonio, San Antonio, TX, USA; 2 College of Nursing and Health Sciences, Texas A&M University-Corpus Christi, Corpus Christi, TX, USA; 3 Department of Sociology and Public Health Program, Florida State University, Tallahassee, FL, USA

**Keywords:** penis size, penis enlargement, guns, firearms, masculinity

## Abstract

In this study, we formally examine the association between penis size dissatisfaction and gun ownership in America. The primary hypothesis, derived from the psychosexual theory of gun ownership, asserts that men who are more dissatisfied with the size of their penises will be more likely to personally own guns. To test this hypothesis, we used data collected from the 2023 *Masculinity, Sexual Health, and Politics* (*MSHAP*) survey, a national probability sample of 1,840 men, and regression analyses to model personal gun ownership as a function of penis size dissatisfaction, experiences with penis enlargement, social desirability, masculinity, body mass, mental health, and a range of sociodemographic characteristics. We find that men who are *more* dissatisfied with the size of their penises are *less* likely to personally own guns across outcomes, including any gun ownership, military-style rifle ownership, and total number of guns owned. The inverse association between penis size dissatisfaction and gun ownership is linear; however, the association is weakest among men ages 60 and older. With these findings in mind, we failed to observe any differences in personal gun ownership between men who have and have not attempted penis enlargement. To our knowledge, this is the first study to formally examine the association between penis size and personal gun ownership in America. Our findings fail to support the psychosexual theory of gun ownership. Alternative theories are posited for the apparent inverse association between penis size dissatisfaction and personal gun ownership, including higher levels of testosterone and constructionist explanations.

## Introduction

Are men with smaller penises more likely to personally own guns than men with larger penises? Although there is no direct empirical evidence linking penis size with personal gun ownership, speculation has been widespread in popular culture. For example, in 2012, a *FOX 31* headline suggested that “Assault rifle owners have ‘tiny penises’” (Holden, 2012). In 2016, an editorial in *HuffPost* claimed that “the compulsion to own firearms stems from an unconscious need to compensate for a deep-seated psychological sense of insecurity and inadequacy in terms of power: in males, specifically for having a small or smaller-than-desired penis” (Blumenfeld, 2016). In 2017, *The Truth About Guns* blog declared that a “Study Confirms Gun Owners Have Smaller Penises” (Zimmerman, 2017). This satirical “study” reported that states with a higher percentage of gun ownership (Kalesan et al., 2016) tended to exhibit lower rates of online purchases of larger-sized condoms (Roy, 2013). In this article, we formally test, for the first time, whether men who are more dissatisfied with the size of their penises are in fact more likely to personally own guns.

The expected association between penis size dissatisfaction and higher rates of gun ownership is derived from what we refer to as the “psychosexual theory of gun ownership” (Hill, Dowd-Arrow, et al., 2021). The theory has four primary propositions. The first proposition is that guns are phallic symbols. Indeed, a symbolic link between guns and male genitalia has persisted for over a century in a range of disciplines, including, for example, African studies, communication studies, feminist studies, gender studies, media studies, porn studies, psychiatry, psychology, and sociology (Blum, 2019; Cooke & Puddifoot, 2000; Diener & Kerber, 1979; Floyd, 2023; Freud, 1922; Hall, 1953; Hill, Dowd-Arrow, Davis, & Burdette, 2020; Kelley, 1995; Moffic, 2013; Nathenson, 2020; Neville-Shepard & Kelly, 2020; Potts, 2000; Sasson-Levy, 2003; Timbs, 2023).

The second proposition is that guns are symbols and instruments of masculinity because they are primarily used by men (Azrael et al., 2017; Burdette et al., 2024; Dowd-Arrow et al., 2019; Ellison, 1991; Goss, 2017; Hepburn et al., 2007; Hill, Wen, et al., 2021; Parker et al., 2017; Smith et al., 2019; Smith & Smith, 1995) and because they can be used to project power and aggressive behavior (Cassino & Besen-Cassino, 2020; Cukier & Eagen, 2018; Cukier & Sheptycki, 2012; Diener & Kerber, 1979; Kahan & Braman, 2003; Nathenson, 2020; Neville-Shepard & Kelly, 2020; Pfaffendorf et al., 2021; Potts, 2000; Stroud, 2012; Tonso, 1982). Stroud (2012, p. 221) explains that “because guns are so lethal, they imbue their users with traits associated with masculinity—control and power.”

The third proposition is when men define their own penises as small or below average, they may experience psychological distress because these perceptions can undermine security, self-confidence, and masculinity (Oates & Sharp, 2017; Sharp et al., 2022; Veale, Miles, Read, et al., 2015; Wylie & Eardley, 2007). Oates and Sharp (2017, p. 1032) explain that “men commonly believe that ‘bigger is better’” because larger penises “symbolize masculinity and sexual prowess.” In this context, “dissatisfaction with penis size has become a leading source of motivation for men to pursue penile augmentation procedures to ultimately increase the length and/or girth of their penis” (Sharp et al., 2022, p. 1306). When men are willing to seek help, they can be motivated by their emotional distress to entertain a variety of enlargement strategies, including, for example, penis pumps, penis weights, stretching exercises, supplements, creams, and surgical procedures (Oates & Sharp, 2017; Sharp et al., 2022; Wylie & Eardley, 2007).

The final proposition of the psychosexual theory of gun ownership suggests that men who are dissatisfied with their penises may seek to obtain guns to compensate for the distressing effects of any perceived deficits in masculinity or sexual potency. The idea is that men who are dissatisfied with the size of their penises are initially attracted to guns because they have been socialized to see guns as symbols of male genitalia and manliness. With this cultural knowledge, some men may be drawn to guns unconsciously (because the loss of masculinity is too painful) or consciously (to express masculinity to themselves and to others). By allowing men “who have felt disempowered to engage with an archetypal symbol of power” (Nathenson, 2020, p. 210), guns may provide some men with a means of psychosexual compensation.

In our review of the literature, we could find only one previous empirical test of the psychosexual theory of gun ownership. Using survey data collected from a national sample of men, Hill, Dowd-Arrow, et al. (2021) examined the association between sexual dysfunction and gun ownership. The key finding was that men who reported experiencing sexual dysfunction exhibited similar rates of gun ownership as men who reported no experiences with sexual dysfunction. This association was replicated across several indicators of sexual dysfunction (performance anxiety, erection trouble, and erectile dysfunction medication) and gun ownership (personal gun ownership, purchasing a gun during the pandemic, and keeping a gun in one’s bedroom). Although this work is informative with respect to sexual dysfunction, the association between penis size and gun ownership has yet to be studied.

In the pages that follow, we use national survey data to extend previous work by directly examining the association between penis size dissatisfaction and personal gun ownership in America. The primary hypothesis, derived from the psychosexual theory of gun ownership, is that men who are more dissatisfied with the size of their penises will be more likely to personally own guns. To test this hypothesis, we examine multiple indicators of penis dissatisfaction (size dissatisfaction and enlargement history) and gun ownership (personal ownership of any gun, personal ownership of a military-style rifle, and the total number of guns owned).

## Data

For this investigation, we use data from the 2023 *Masculinity, Sexual Health, and Politics (MSHAP)* survey. The primary purpose of the *MSHAP* survey is to empirically document the intersection of masculinity, sexual health, and politics in the United States. More specifically, the *MSHAP* survey is based on a national probability sample of 2,024 community-dwelling men aged 18 and over living in the United States. Respondents were sampled from the National Opinion Research Center’s (NORC) *AmeriSpeak* panel, which is representative of households from all 50 states and the District of Columbia (AmeriSpeak, 2022). Sampled respondents were invited to complete an online survey in English between March 30, 2023 and April 12, 2023. The data collection process yielded a weighted cumulative response rate of 4.7%. The weighted cumulative response rate, which considers all panel recruitment and retention rates, is the overall survey response rate that accounts for survey outcomes in all response stages, including the panel recruitment rate, panel retention rate, and survey completion rate. It is weighted to account for the sample design and differential inclusion probabilities of sample members. Our cumulative response rate is within the range (4%–5%) typically reported by high-quality general population surveys (see Pew Research Center, 2021). The multistage probability sample resulted in a margin of error of ±3.08% and an average design effect of 2.00. Margin of error is defined as half the width of the 95% confidence interval for a proportion estimate of 50% adjusted for design effect. A figure of ±3.08% is therefore the largest margin of error possible for all estimated percentages based on the study sample. A margin of error of ±3.08% at the 95% confidence level means that if we fielded the same survey 100 times, we would expect the result to be within 3.08% of the true population value 95 times. A margin of error of 3.00 is considered very good (Cui, 2002). The average design effect is the variance under the complex design divided by the variance under a simple random sampling design of the same sample size. The design effect is variable-specific and the reported value is the average design effect calculated for a set of key survey variables. Design effects account for deviations from simple random sampling with a 100% response rate. A design effect of 2.00 is very good because it means that the variance is only about twice as large as would be expected with simple random sampling (Kish, 1965). The median self-administered web-based survey lasted approximately 10 minutes. All respondents were offered the cash equivalent of $3.00 for completing the survey. The survey was reviewed and approved by the institutional review boards at NORC and the University of Texas at San Antonio (IRB #: FY22-23-196). Written informed consent was obtained from all participants.

## Measures

### Gun Ownership

Gun ownership is measured with three items: (a) whether a respondent personally owns “any guns or firearms” (1 = *yes*; 0 = *no*), (b) whether a respondent personally owns “any semi-automatic or fully automatic military-style rifles, such as AR-15, AK-47, or SCAR” (1 = *yes*; 0 = *no*), and (c) the total number of “guns or firearms” the respondent personally owns (top-coded 0 to 5 or more).

### Penis Size Dissatisfaction

We measure penis size dissatisfaction in two ways. To assess *penis size dissatisfaction*, respondents were asked to indicate their overall level of dissatisfaction with the size of their penis when fully erect (1 = *completely satisfied* to 7 = *completely dissatisfied*) (Veale et al., 2014). To assess experiences with penis enlargement, respondents were asked to indicate whether they had ever used any methods for penis enlargement, such as penis pumps, penis weights, stretching exercises, supplements, creams, or surgeries (1 = *ever used any methods of penis enlargement*; 0 = *never used any methods of penis enlargement*).

### Potential Correlates of Penis Size Dissatisfaction

While penis size dissatisfaction has been linked with penis appearance dissatisfaction, dissatisfaction with one’s sex life, and greater body mass (Lever et al., 2006; Sharp et al., 2022; Veale et al., 2014; Veale, Miles, Bramley, et al., 2015; Veale, Miles, Read, et al., 2015), penis size dissatisfaction is inconsistently associated with or unrelated to number of sex partners and psychological distress (Lee, 1996; Sharp et al., 2022). Following this research, we assess the construct validity of our focal predictor variables by testing several potential correlates of penis size dissatisfaction. We measure *penis appearance dissatisfaction* by asking respondents to indicate their overall level of satisfaction with the appearance of their penis when fully erect (1 = *completely satisfied* to 7 = *completely dissatisfied*) (Veale et al., 2014). To measure *sex life dissatisfaction*, we asked respondents to indicate their level of dissatisfaction with their overall sex life (1 = *dissatisfied*; 0 = *neither satisfied or dissatisfied or satisfied*). *Number of sex partners* was measured in the past 12 months (top-coded 0 to 3 or more). *Obesity* was measured with self-reports of height and weight (body mass index ≥30, weight (lb)/(self-reported height [in])2 × 703). Our measurement of overall mental health status asked respondents to rate their general mental health (1 = *poor* to 5 = *excellent*) (Ahmad et al., 2014). *Masculinity* was indicated by self-rated manliness (1 = not at all “manly” to 10 = very “manly”). Finally, we measured *social desirability* as a summed index of 7 items (e.g., “I am always courteous, even to people who are disagreeable.” “I sometimes feel resentful when I don’t get my way.”) (Fischer & Fick, 1993).

### Background Variables

Multivariate analyses include several potential background correlates of penis dissatisfaction and gun ownership, including *age* (dummy variables for 30–44, 45–59, and 60–95, with 18–29 serving as the reference category), *sexuality* (1 = *straight*, that is, not gay; 0 = *gay or bisexual*), *race/ethnicity* (1 = *non-Hispanic White*; 0 = *otherwise*), *nativity* (1 = *born in the United States*; 0 = *otherwise*), *education* (1 = *4-year college degree or higher*; 0 = *otherwise*), *employment status* (1 = *employed for pay*; 0 = *otherwise*), *household income* (1 = *<$10,000* to 9 = *≥$150,000*), *marital status* (1 = *married*; 0 = *otherwise*), *rural residence* (1 = *nonmetropolitan area*; 0 = *metropolitan area*), and *southern residence* (1 = *southern state*; 0 = *otherwise*).

## Analysis

After using listwise deletion for missing data, our analytic sample size was reduced from 2,024 to 1,840. In other words, over 90% of the total possible sample was retained across regression models. Most of the missing data were attributed to nonresponses to self-reported height and weight (*n* = 100), number of sex partners (*n* = 100), penis appearance dissatisfaction (*n* = 78), penis enlargement (*n* = 76), and penis size dissatisfaction (*n* = 62).

Poststratification weights were used to address sampling error and nonresponse bias. NORC developed poststratification weights for *MSHAP* via iterative proportional fitting or raking to general population parameters derived from the Current Population Survey (2022). These parameters included age, gender, census region, race/ethnicity, education, housing tenure, household phone status, and the interaction of age and gender (age*gender).

Subsequent analyses begin with weighted descriptive statistics for all study variables, including variable ranges, sample means, and standard deviations (Table 1). Next, to assess the construct validity of our focal predictors, we model penis size dissatisfaction and penis enlargement as a function of penis appearance dissatisfaction, sex life dissatisfaction, number of sex partners, obesity, mental health, masculinity, social desirability, and background variables (Table 2). We then use binary logistic regression to model any gun ownership (Table 3) and military-style rifle ownership (Table 4). We also use negative binomial regression to model the count of total guns owned (Table 5). Our focal regressions include 4 models. Model 1 regresses gun ownership on penis size dissatisfaction and penis enlargement. Model 2 adds background variables to Model 1. To assess the linearity of the association between penis size dissatisfaction and gun ownership, Model 3 adds a penis size dissatisfaction squared term to Model 2. Finally, to test whether the association between penis size dissatisfaction and gun ownership varies by age, Model 4 adds interaction terms (penis size dissatisfaction*age categories) to Model 2.

**Table 1.** Weighted Descriptive Statistics (MSHAP 2023)

Variable	Range	*M*	*SD*
Any gun owner	0–1	0.43	
Military-style rifle owner	0–1	0.11	
Total guns owned	0–5+	1.15	
Penis size dissatisfaction	1–7	2.63	0.05
Penis enlargement	0–1	0.07	
Penis appearance dissatisfaction	1–7	2.43	0.05
Sex life dissatisfaction	0–1	0.30	
Number of sex partners	0–3+	0.93	0.03
Obese	0–1	0.34	
Self-rated mental health	1–5	3.51	0.03
Social desirability	0–7	4.02	0.05
Ages 18–29	0–1	0.19	
Ages 30–44	0–1	0.27	
Ages 45–59	0–1	0.24	
Ages 60–95	0–1	0.30	
Straight sexuality	0–1	0.92	
Masculinity	1–10	8.08	0.06
Non-Hispanic White	0–1	0.64	
Non-Hispanic Black	0–1	0.10	
Latino	0–1	0.18	
Asian	0–1	0.06	
Other race/ethnicity	0–1	0.03	
U.S.-born	0–1	0.91	
College degree	0–1	0.36	
Employed	0–1	0.65	
Household income	1–9	6.16	0.08
Married	0–1	0.55	
Rural residence	0–1	0.14	
Southern residence	0–1	0.37	

*Note. n* = 1,840.

**Table 2.** Weighted Regression of Penis Size Dissatisfaction and Enlargement (MSHAP 2023)

Variable	Penis size dissatisfaction	Penis enlargement
Penis appearance dissatisfaction	0.84*** (0.02)	1.27*** (1.12, 1.45)
Sex life dissatisfaction	0.16* (0.07)	1.89 (0.99, 3.59)
Number of sex partners	−0.01 (0.05)	1.65** (1.15, 2.38)
Obese	0.22** (0.06)	1.28 (0.70, 2.33)
Self-rated mental health	0.03 (0.03)	1.31* (1.01, 1.69)
Masculinity	0.007 (0.02)	0.87 (0.73, 1.03)
Social desirability	−0.003 (0.02)	0.85 (0.70, 1.01)
Ages 30–44	−0.19 (0.12)	1.93 (0.75, 5.03)
Ages 45–59	−0.18 (0.11)	1.42 (0.52, 3.84)
Ages 60–95	0.001 (0.11)	1.63 (0.55, 4.87)
Straight sexuality	−0.09 (0.09)	0.56 (0.24, 1.31)
Non-Hispanic white	−0.02 (0.07)	0.71 (0.36, 1.40)
U.S.-born	−0.03 (0.11)	0.82 (0.18, 3.82)
College degree	−0.10 (0.06)	0.50 (0.23, 1.08)
Employed	0.01 (0.08)	0.55 (0.28, 1.09)
Household income	0.01 (0.02)	0.84* (0.72, 0.97)
Married	0.05 (0.08)	1.15 (0.52, 2.54)
Rural residence	0.004 (0.08)	0.93 (0.42, 2.02)
Southern residence	0.04 (0.07)	1.20 (0.66, 2.21)

*Notes: n* = 1,840. Penis size dissatisfaction includes unstandardized coefficients and standard errors obtained from a weighted OLS regression. Penis enlargement includes odds ratios and 95% confidence intervals obtained from a weighted binary logistic regression. The reference category for age is 18–29.

* *p* < .05. **p < .01. ***p < .001.

**Table 3.** Weighted Binary Logistic Regression of Any Gun Ownership (MSHAP 2023)

Variable	Model 1	Model 2	Model 3	Model 4
Penis size dissatisfaction	0.91* (0.84, 0.99)	0.89* (0.81, 0.97)	0.92 (0.81, 1.05)	0.69**(0.54, 0.88)
Penis size dissatisfaction squared			0.98 (0.93, 1.03)	
Penis enlargement	0.99 (0.56, 1.76)	1.44 (0.82, 2.53)	1.44 (0.82, 2.53)	1.49 (0.83, 2.66)
Ages 30–44		2.36*** (1.48, 3.76)	2.37*** (1.49, 3.78)	2.55*** (1.58, 4.11)
Ages 45–59		4.24*** (2.61, 6.90)	4.25*** (2.61, 6.92)	4.52*** (2.74, 7.47)
Ages 60–95		5.24*** (3.20, 8.55)	5.19*** (3.18, 8.47)	5.62*** (3.42, 9.22)
Size dissatisfaction × ages 30–44				1.33* (1.00, 1.76)
Size dissatisfaction × ages 45–59				1.21 (0.90, 1.64)
Size dissatisfaction × ages 60–95				1.40* (1.06, 1.85)
Straight Sexuality		3.15**(1.64, 6.03)	3.15**(1.64, 6.02)	3.23*** (1.65, 3.33)
Non-Hispanic white		2.34*** (1.65, 3.33)	2.35*** (1.65, 3.34)	2.35*** (1.65, 3.33)
U.S.-born		1.96* (1.06, 3.60)	1.95* (1.05, 3.59)	2.02* (1.10, 3.71)
College degree		0.83 (0.60, 1.16)	0.83 (0.59, 1.16)	0.81 (0.58, 1.13)
Employed		1.15 (0.81, 1.64)	1.15 (0.80, 1.64)	1.17 (0.82, 1.66)
Household income		1.11**(1.03, 1.19)	1.11**(1.03, 1.19)	1.11**(1.03, 1.20)
Married		0.98 (0.72, 1.35)	0.99 (0.72, 1.36)	0.97 (0.71, 1.33)
Rural residence		2.24*** (1.50, 3.34)	2.24*** (1.50, 3.35)	2.19*** (1.47, 3.27)
Southern residence		1.65**(1.23, 2.22)	1.65** (1.22, 2.21)	1.70*** (1.26, 2.29)
Obese		1.42* (1.05, 1.92)	1.41* (1.04, 1.92)	1.44* (1.06, 1.96)
Self-rated mental health		1.11 (0.95, 1.29)	1.11 (0.95, 1.30)	1.11 (0.95, 1.29)
Masculinity		1.16**(1.05, 1.27)	1.16**(1.05, 1.28)	1.15**(1.04, 1.27)
Social desirability		0.93 (0.84, 1.02)	0.93 (0.85, 1.02)	0.93 (0.84, 1.02)

*Note. n* = 1,840. Shown are odds ratios and 95% confidence intervals obtained from a series of weighted binary logistic regressions. The reference category for age is 18–29.

* *p* < .05. **p < .01. ***p < .001.

**Table 4.** Weighted Binary Logistic Regression of Military-Style Rifle Ownership (MSHAP 2023)

Variable	Model 1	Model 2	Model 3	Model 4
Penis size dissatisfaction	0.87* (0.77, 0.99)	0.80** (0.68, 0.94)	0.80* (0.66, 0.97)	0.61* (0.42, 0.90)
Penis size dissatisfaction squared			1.00 (0.93, 1.08)	
Penis enlargement	1.40 (0.69, 2.85)	1.20 (0.54, 2.66)	1.20 (0.54, 2.66)	1.26 (0.57, 2.80)
Ages 30–44		2.45* (1.24, 4.88)	2.45* (1.24, 4.88)	2.83** (1.40, 5.71)
Ages 45–59		1.87 (0.90, 3.86)	1.87 (0.90, 3.85)	1.67 (0.78, 3.58)
Ages 60–95		1.49 (0.65, 3.43)	1.49 (0.65, 3.42)	1.68 (0.73, 3.87)
Size dissatisfaction × ages 30–44				1.43 (0.94, 2.19)
Size dissatisfaction × ages 45–59				0.88 (0.51, 1.52)
Size dissatisfaction × ages 60–95				1.68* (1.10, 2.56)
Straight sexuality		3.70* (1.23, 11.19)	3.70* (1.23, 11.17)	3.76* (1.25, 11.33)
Non-Hispanic white		1.50 (0.88, 2.58)	1.50 (0.88, 2.58)	1.52 (0.89, 2.61)
U.S.-born		0.84 (0.34, 2.09)	0.84 (0.34, 2.08)	0.88 (0.35, 2.18)
College degree		0.46** (0.28, 0.76)	0.46** (0.28, 0.77)	0.44** (0.27, 0.73)
Employed		0.99 (0.56, 1.77)	0.99 (0.56, 1.76)	1.03 (0.59, 1.79)
Household income		1.13 (0.99, 1.28)	1.13 (0.99, 1.27)	1.14* (1.01, 1.28)
Married		0.83 (0.49, 1.38)	0.82 (0.49, 1.38)	0.82 (0.49, 1.35)
Rural residence		1.54 (0.92, 2.58)	1.54 (0.92, 2.58)	1.49 (0.89, 2.47)
Southern residence		0.98 (0.64, 1.49)	0.98 (0.64, 1.49)	1.03 (0.68, 1.57)
Obese		1.86** (1.21, 2.86)	1.86** (1.21, 2.87)	1.93** (1.26, 2.93)
Self-rated mental health		1.00 (0.79, 1.27)	1.00 (0.79, 1.27)	1.01 (0.80, 1.28)
Masculinity		1.05 (0.90, 1.22)	1.05 (0.90, 1.22)	1.05 (0.90, 1.22)
Social desirability		0.87* (0.77, 0.99)	0.87* (0.77, 0.99)	0.87* (0.76, 0.98)

*Note. n* = 1,840. Shown are odds ratios and 95% confidence intervals obtained from a series of weighted binary logistic regressions. *p < .05, **p < .01, ***p < .001. The reference category for age is 18–29.

* *p* < .05. **p < .01. ***p < .001.

**Table 5.** Weighted Negative Binomial Regression of Total Gun Ownership (MSHAP 2023)

Variable	Model 1	Model 2	Model 3	Model 4
Penis size dissatisfaction	0.92** (0.86, 0.98)	0.89** (0.83, 0.95)	0.90* (0.82, 0.98)	0.64*** (0.52, 0.80)
Penis size dissatisfaction squared			0.99 (0.96, 1.02)	
Penis enlargement	0.79 (0.52, 1.20)	0.92 (0.60, 1.43)	0.92 (0.60, 1.42)	1.00 (0.64, 1.56)
Ages 30–44		2.22*** (1.53, 3.21)	2.22*** (1.53, 3.23)	2.49*** (1.73, 3.58)
Ages 45–59		3.25*** (2.23, 4.74)	3.26*** (2.23, 4.75)	3.55*** (2.46, 5.13)
Ages 60–95		3.22*** (2.19, 4.74)	3.20*** (2.18, 4.70)	3.56*** (2.46, 5.13)
Size dissatisfaction × ages 30–44				1.41** (1.10, 1.80)
Size dissatisfaction × ages 45–59				1.25 (0.97, 1.61)
Size dissatisfaction × ages 60–95				1.58*** (1.25, 2.00)
Straight sexuality		2.66** (1.51, 4.67)	2.66**(1.52, 4.66)	2.70** (1.53, 4.77)
Non-Hispanic white		1.69*** (1.30, 2.20)	1.70*** (1.30, 2.21)	1.71*** (1.32, 2.22)
U.S.-born		1.66* (1.04, 2.66)	1.65* (1.03, 2.65)	1.74* (1.09, 2.77)
College degree		0.77* (0.62, 0.96)	0.77* (0.62, 0.96)	0.76* (0.61, 0.95)
Employed		1.06 (0.84, 1.34)	1.05 (0.83, 1.33)	1.09 (0.87, 1.36)
Household income		1.09** (1.03, 1.15)	1.09** (1.03, 1.15)	1.09** (1.04, 1.15)
Married		1.02 (0.83, 1.26)	1.02 (0.83, 1.26)	1.01 (0.82, 1.24)
Rural residence		1.56*** (1.22, 2.01)	1.57*** (1.22, 2.01)	1.53** (1.20, 1.95)
Southern residence		1.17 (0.95, 1.44)	1.17 (0.95, 1.44)	1.21 (0.99, 1.48)
Obese		1.24* (1.00, 1.54)	1.24* (1.00, 1.54)	1.27* (1.03, 1.57)
Self-rated mental health		1.04 (0.93, 1.16)	1.04 (0.93, 1.17)	1.04 (0.93, 1.16)
Masculinity		1.05 (0.97, 1.14)	1.06 (0.97, 1.14)	1.06 (0.98, 1.14)
Social desirability		0.90** (0.84, 0.97)	0.90 (0.84, 0.97) **	0.90** (0.84, 0.97)

*Note. n* = 1,840. Shown are incidence rate ratios (IRR) and 95% confidence intervals obtained from a series of weighted negative binomial regressions. The reference category for age is 18–29.

* *p* < .05. **p < .01. ***p < .001.

## Results

### Descriptive Analyses

Table 1 shows that 43% (*n* = 791) of men reported personal ownership of a gun, and 11% (*n* = 202) reported ownership of a military-style rifle. The average respondent owned approximately one gun. Respondents reported low levels of dissatisfaction with penis size and penis appearance, and only 7% (*n* = 129) of men reported having used a method of penis enlargement. While 30% (*n* = 552) of men reported being dissatisfied with their overall sex lives, the average respondent reported having approximately one sex partner in the past year.

### Construct Validity

Table 2 assesses the construct validity of penis size dissatisfaction and penis enlargement. Our analyses revealed several associations that are consistent with previous research. Men who were more dissatisfied with the overall appearance of their penises tended to be more dissatisfied with the size of their penises (*b* = 0.84, *p* < .001) and were more likely to have attempted penis enlargement (OR = 1.27, *p* < .001). Men who were dissatisfied with their sex lives (*b* = .16, *p* < .05) and obese men (*b* = .22, *p* < .01) tended to be more dissatisfied with the size of their penises. While men who reported having more sex partners in the past year (OR = 1.65, *p* < .01) and better mental health (OR = 1.31, *p* < .05) were more likely to have attempted penis enlargement, men who reported greater household incomes were less likely to have attempted enlargement (OR = .84, *p* < .05). Finally, penis size dissatisfaction and penis enlargement were unrelated to masculinity, social desirability, age, sexual orientation, race/ethnicity, nativity status, education, employment, marital status, rural residence, and southern residence.

### Gun Ownership

Tables 3 to 5 feature the regression models for gun ownership. The odds ratios reported in Tables 3 and 4 describe the difference in the expected odds of personally owning a gun or a military-style rifle for each one-unit change in a predictor. The incidence rate ratios (IRR) reported in Table 5 are interpreted as the difference in the expected count of total guns owned for each one-unit change in a predictor. According to Model 2 of Tables 3 and 4, the odds of owning a gun (any gun or a military-style rifle) are *lower* for men who are *more* dissatisfied with the size of their penises. In fact, each one-unit increase in penis size dissatisfaction *reduces* the odds of owning any gun by 11% (OR = 0.89, *p* < .05) and the odds of owning a military-style rifle by 20% (OR = .80, *p* < .01). According to Model 2 of Table 5, each one-unit increase in penis size dissatisfaction also *reduces* the expected count of total guns owned by 11% (IRR = 0.89, *p* < .01). Across outcomes, we failed to observe any associations between penis enlargement and gun ownership.

Model 3 of Tables 3 to 5 assesses the linearity of the association between penis size dissatisfaction and gun ownership. Across outcomes, the squared term for penis size dissatisfaction was not different from zero. These null findings suggest linear associations between penis size dissatisfaction and gun ownership outcomes.

**Figure 1.**
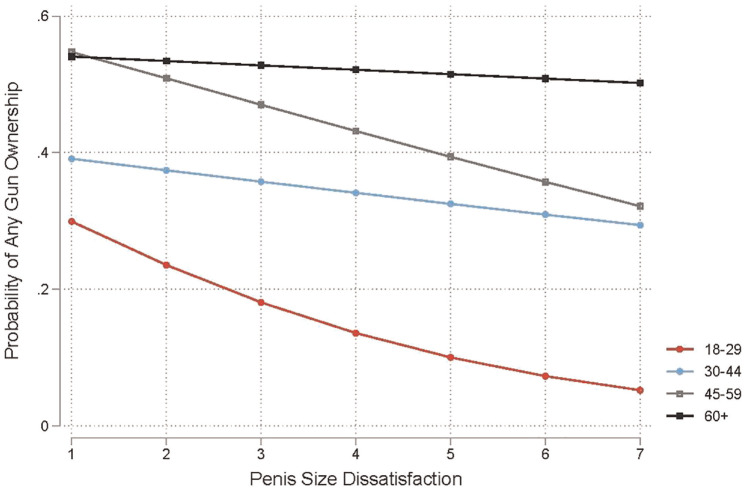
Probability of Any Gun Ownership by Penis Size Dissatisfaction and Age

**Figure 2.**
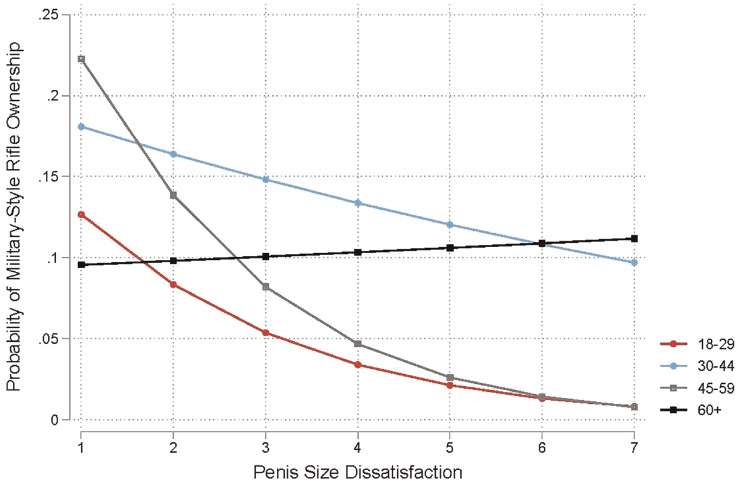
Probability of Military-Style Rifle Ownership by Penis Size Dissatisfaction and Age

**Figure 3.**
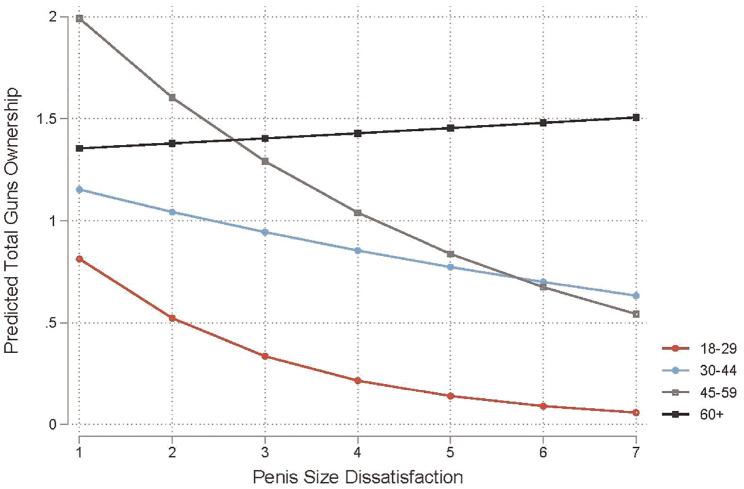
Predicted Total Gun Ownership by Penis Size Dissatisfaction and Age

Model 4 of Tables 3 to 5 tests whether the association between penis size dissatisfaction and gun ownership varies by age. Across outcomes, we find that the association between penis size dissatisfaction and gun ownership is stronger for younger men (ages 18–29) than for older men (ages 60 and older). We also observe some nonlinear moderation patterns by age. For any gun ownership and total guns owned, the association between penis size dissatisfaction and gun ownership is stronger for men ages 18 to 29 than for men ages 30 to 44. In the case of military-style rifle ownership, the association between penis size dissatisfaction and gun ownership is comparable for men ages 18 to 29 and men ages 30 to 44. Interestingly, across outcomes, the association between penis size dissatisfaction and gun ownership is comparable for men ages 18 to 29 and men ages 45 to 59. Figures 1 to 3 illustrate the steepest negative slopes for penis size dissatisfaction among men ages 18 to 29 and 45 to 59. In contrast, the negative slopes are slightly attenuated for men ages 30 to 44. We observe the weakest associations between penis size dissatisfaction and gun ownership among men ages 60 and older. In fact, the associations for this group are mostly null (flat) across gun ownership outcomes.

Although our focus is on penis size, we noted several other consistent associations with gun ownership. Across outcomes, straight men and obese men tend to exhibit higher rates of gun ownership. Older men, U.S.-born men, men who report greater household income, and rural residents are also more likely to own any gun (but not a military-style rifle) and to own more guns. Men with college degrees are less likely to own military-style rifles and tend to report owning fewer guns. Interestingly, men who score higher on social desirability are less likely to report owning a military-style rifle (not any gun) and tend to report owning fewer guns. Finally, men who live in the south and score higher on masculinity are more likely report any gun ownership.

## Discussion

Although the association between penis size and personal gun ownership has been a persistent topic in popular culture, it has escaped any direct empirical analysis. In this article, we formally tested whether men who are more dissatisfied with the size of their penises are in fact more likely to personally own guns in a national sample of American men. The primary hypothesis, derived from the psychosexual theory of gun ownership, stated that men who are more dissatisfied with the size of their penises would be more likely to personally own guns. Our analyses consistently failed to support the hypothesis. Instead, we found that rates of gun ownership were similar for men who had attempted penis enlargement and men who had no experiences with penis enlargement. We also observed that men who were *less* dissatisfied with the size of their penises were *more* likely to personally own guns across outcomes, including any gun ownership, military-style rifle ownership, and the total number of guns owned. To our knowledge, this is the first study to formally test the association between penis size dissatisfaction and personal gun ownership in America.

Given that the psychosexual theory of gun ownership attempts to link small penis size with higher rates of personal gun ownership, it is unclear why men who are less dissatisfied with the size of their penises would be more likely to personally own guns. The reported age variations suggest one of two plausible theories for future research. While the association between penis size dissatisfaction and gun ownership was strongest among men ages 18 to 29 and 45 to 59, it was weakest among men ages 60 and older. On one hand, these patterns may be interpreted through the findings of research on the age-patterning of testosterone in men. For example, Kanabar and colleagues (2022, p. 29) report “a steep decline” in testosterone “around age 30” and a “rebound around age 50.” These patterns are important because greater testosterone levels tend to favor larger penises (Bin-Abbas et al., 1999; Boas et al., 2006; de Castro Paiva et al., 2016) and greater risk-taking and social dominance behavior (Booth et al., 2006; Geniole & Carré, 2018; Willer et al., 2013; Zitzmann, 2020). Willer and colleagues (2013, p. 986) note that higher testosterone levels are consistently associated with more “masculine behaviors and attitudes related to dominance, aggression, power, risk-taking, and competitiveness.” In this context, the apparent inverse association between penis size dissatisfaction and personal gun ownership could be spurious due to the omission of testosterone levels.

On the other hand, social constructionist theories of gender recognize that cultural definitions and enactments of masculinity vary dramatically across the life span and may take the form of “hybrid masculinities” animated by diverse (even contradictory) social forces (e.g., Diefendorf, 2015). Therefore, it is possible that men think quite differently about manhood, their bodies (including its sexual apparatus), and gender-resonant symbols such as guns before, say, age thirty than in later life. These perceptions may be culturally conditioned among men as they make their “gender journey” through life (Todd et al., 2022). So, beyond biological factors, penis size and its connection to guns may be more salient for younger men than older men, given cultural connections that resonate with young men still seeking to establish themselves in life. In our data, young gun-owning men are not compensating for what they perceive to be small penises. But this does not rule out guns as a potentially potent gender symbol. It is possible that young gun-owning men have a penile preoccupation (albeit expressed as size satisfaction) that is not found in older men or their nonfirearm-owning younger peers. These latter groups may express less satisfaction because they simply do not care as much about their penis size (thus, a degree of penile apathy). So, one cannot rule out sexual fixation as a possible influence in gun ownership. In short, more research is needed to distinguish penis size satisfaction from a broader penile preoccupation, a social construct that may contribute to perceptions of size and to gun ownership at younger ages.

The findings reported here are notable because they provide a novel test of the psychosexual theory of gun ownership. Although our results for penis enlargement are consistent with a previous study of sexual dysfunction and personal gun ownership (Hill, Dowd-Arrow, et al., 2021), our results for penis size dissatisfaction are unprecedented. Whether penis size dissatisfaction limits gun ownership or is unrelated to gun ownership, the psychosexual theory of gun ownership remains unsupported. Guns are clearly phallic symbols. Guns are clearly associated with masculinity. However, in our analyses, penis size dissatisfaction is unrelated to mental health. The psychosexual theory of gun ownership consistently fails in its assertion that men who have trouble with their penises or are dissatisfied with their penises are especially likely to acquire guns as a means of compensation. While some arguments for “compensatory masculinity” are quite credible (e.g., violence as a means of asserting masculinized power in a virulent and criminal fashion), this study cautions against universalizing this theoretical construct to include all risky behavior (Courtenay, 2000). Gun ownership is more likely driven by greater masculinity, diverse constructs of manhood, or perhaps even higher levels of testosterone than by deficits in masculinity or sexual health.

Although our findings suggested that social desirability was unrelated to penis size dissatisfaction, it was associated with lower rates of military-style rifle ownership and fewer total guns owned. Urbatsch (2019, p. 190) explains that social desirability bias “can occur when respondents want to veil answers that they suspect the survey takers will dislike.” Our findings suggest that some gun owners may be “reluctant to answer questions” because they are “stigmatized—or perceive themselves to be stigmatized” for their gun ownership (Urbatsch, 2019, p. 190). It is interesting that while social desirability bias was observed when respondents were asked about owning “semi-automatic or fully automatic military-style rifles, such as AR-15, AK-47, or SCAR” or about the total number of “guns or firearms” they personally own, no social desirability bias was observed for the generic question of whether the respondent personally owns “any guns or firearms.” Our results suggest that, even when self-administered web-based surveys are employed to limit social desirability bias, men may systematically underestimate certain types of gun ownership when they are asked questions that could evoke stigmatizing images of “mass shootings,” “arsenals,” or “stockpiles.”

Finally, we acknowledge that our analyses are limited in two key respects. First, because this analysis is based on a cross-sectional design, no causal or temporal inferences can be made. Second, because penis dissatisfaction is measured with single-item self-reports, it is important for future research to develop more reliable multi-item indices and to consider more valid and direct measurements of penis size. With this limitation in mind, it is important to note that any associations involving penis dissatisfaction are likely to be underestimated because measurement error tends to undermine statistical power (i.e., our ability to detect statistically significant differences in the population) (Kanyongo et al., 2007).

## Conclusion

Our analyses show that men who are less dissatisfied with the size of their penises are more likely to own guns than other men. These findings are important because they contribute to an evidence-based understanding of gun ownership. Gun owners make a lot of claims about guns. Many will tell you that guns improve their lives, make them happy, and help them sleep better at night, but none of these claims have been established empirically (Hill, Dowd-Arrow, Burdette, & Hale, 2020; Hill, Dowd, Arrow, Burdette, & Warner, 2020; Hill, Dowd-Arrow, Davis, & Burdette, 2020). People who do not own guns will tell you that gun owners are motivated by fear or sexual dysfunction, but these ideas are also unfounded (DeFronzo, 1979; Dowd-Arrow et al., 2019; Hauser & Kleck, 2013; Hill, Dowd-Arrow, et al., 2021; Kleck, 1997). In these instances, gun culture rhetoric functions to justify guns (guns are personally helpful), discredit gun owners (gun owners are compensators), and further stigmatize men with smaller penises. Ultimately, these kinds of discussions are counterproductive for society because they distract us from the observable realities of penis dissatisfaction and gun ownership. We know that guns threaten public health in the United States (Fleegler et al., 2013; Fowler et al., 2015; Gani et al., 2017; Gramlich, 2019; Miller et al.,2002, 2007; Spitzer et al., 2017; Van Kesteren, 2014). We also know that men who are dissatisfied with their penises also tend to exhibit lower levels of personal well-being (Oates & Sharp, 2017; Sharp et al., 2022; Veale, Miles, Read, et al., 2015; Wylie & Eardley, 2007). It is clear that both of these issues will persist until we commit ourselves to more evidence-based discussions of the taken-for-granted assumptions of the role of guns in society.

## References

[bibr1-15579883241255830] Ahmad F. Jhajj A. K. Stewart D. E. Burghardt M. Bierman A. S. (2014). Single item measures of self-rated mental health: A scoping review. BMC Health Services Research, 14, 1–11. 25231576 10.1186/1472-6963-14-398 PMC4177165

[bibr2-15579883241255830] AmeriSpeak. (2022). Technical overview of the AmeriSpeak® panel NORC’s probability-based household panel. University of Chicago. https://amerispeak.norc.org/Documents/Research/AmeriSpeak%20Technical%20Overview%202019%2002%2018.pdf

[bibr3-15579883241255830] Azrael D. Hepburn L. Hemenway D. Miller M. (2017). The stock and flow of firearms: Results from the 2015 National Firearms Survey. RSF: Russell Sage Foundation Journal of the Social Sciences, 3, 38–57.

[bibr4-15579883241255830] Bin-Abbas B. Conte F. Grumbach M. Kaplan S. (1999). Congenital hypogonadotropic hypogonadism and micropenis: Effect of testosterone treatment on adult penile size—Why sex reversal is not indicated. The Journal of Pediatrics, 134, 579–583. 10228293 10.1016/s0022-3476(99)70244-1

[bibr5-15579883241255830] Blum L. (2019, October 13). What guns often protect is a sense of manhood. Psychology Today. https://www.psychologytoday.com/us/blog/beyond-freud/201910/what-guns-often-protect-is-sense-manhood

[bibr6-15579883241255830] Blumenfeld W. (2016, October 12). Gun control debate: A view from Hoplophiliaville. The Huffington Post. https://www.huffpost.com/entry/gun-control-debate-a-view_b_8274902

[bibr7-15579883241255830] Boas M. Boisen K. Virtanen H. Kaleva M. Suomi A. Schmidt I. . . .Main K. (2006). Postnatal penile length and growth rate correlate to serum testosterone levels: A longitudinal study of 1962 normal boys. European Journal of Endocrinology, 154, 125–129. 16382001 10.1530/eje.1.02066

[bibr8-15579883241255830] Booth A. Granger D. Mazur A. Kivlighan K. (2006). Testosterone and social behavior. Social Forces, 85, 167–191.

[bibr9-15579883241255830] Burdette A. Lawrence E. Hill T. Taylor M. Dowd-Arrow B. (2024). Do Men and Women Integrate Guns into Risky Health Lifestyles in Young Adulthood? Sociological Inquiry, 94, 290–307.

[bibr10-15579883241255830] Cassino D. Besen-Cassino Y. (2020). Sometimes (but not this time), a gun is just a gun: Masculinity threat and guns in the United States, 1999–2018. Sociological Forum, 35, 5–23.

[bibr11-15579883241255830] Cooke C. Puddifoot J. (2000). Gun culture and symbolism among U.K. and U.S. women. The Journal of Social Psychology, 140, 423–433. 10981372 10.1080/00224540009600482

[bibr12-15579883241255830] Courtenay W. (2000). Constructions of masculinity and their influence on men’s well-being: A theory of gender and health. Social Science & Medicine, 50, 1385–1401. 10741575 10.1016/s0277-9536(99)00390-1

[bibr13-15579883241255830] Cui W. (2002). Reducing error in mail surveys. Practical Assessment, Research, and Evaluation, 8, 18.

[bibr14-15579883241255830] Cukier W. Eagen S. (2018). Globalization of gun culture transnational reflections on pistolization and masculinity, flows and resistance. International Journal of Law, Crime and Justice, 40, 3–19.

[bibr15-15579883241255830] Cukier W. Sheptycki J. (2012). Gun violence. Current Opinion in Psychology, 19, 109–112. 10.1016/j.copsyc.2017.04.008 29279206

[bibr16-15579883241255830] Current Population Survey. (2022). Current population survey data. U.S. Census Bureau. https://www.census.gov/programs-surveys/cps/data.html

[bibr17-15579883241255830] de Castro Paiva K. Bastos A. Miana L. de Souza Barros E. Ramos P. Miranda L. . . . Netto J . (2016). Biometry of the hypospadic penis after hormone therapy (testosterone and estrogen): A randomized, double-blind controlled trial. Journal of Pediatric Urology, 12, 200.e1–200.e6. 10.1016/j.jpurol.2016.04.013 27321554

[bibr18-15579883241255830] DeFronzo J. (1979). Fear of crime and handgun ownership. Criminology, 17, 331–340.

[bibr19-15579883241255830] Diefendorf S. (2015). After the wedding night: Sexual abstinence and masculinities over the life course. Gender & Society, 29, 647–669.

[bibr20-15579883241255830] Diener E. Kerber K. (1979). Personality characteristics of American gun-owners. The Journal of Social Psychology, 107, 227–238. 28135439 10.1080/00224545.1979.9922703

[bibr21-15579883241255830] Dowd-Arrow B. Hill T. Amy Burdette A. (2019). Gun ownership and fear. SSM-population Health, 8, 100463. 31414039 10.1016/j.ssmph.2019.100463 PMC6687226

[bibr22-15579883241255830] Ellison C. (1991). Southern culture and gun ownership. Social Science Quarterly, 72, 267–283.

[bibr23-15579883241255830] Fischer D. Fick C. (1993). Measuring social desirability: Short forms of the Marlowe- Crowne social desirability scale. Educational and Psychological Measurement, 53, 417–424.

[bibr24-15579883241255830] Fleegler E. Lee L. Monuteaux M. Hemenway D. Mannix R. (2013). Firearm legislation and firearm-related fatalities in the United States. JAMA Internal Medicine, 173, 732–740. 23467753 10.1001/jamainternmed.2013.1286

[bibr25-15579883241255830] Floyd J. (2023). “The words which sailor John put to them when unrestrained were the veriest filth”: Situating chanteys in the field of porn studies. Porn Studies, 10, 8–19.

[bibr26-15579883241255830] Fowler K. Dahlberg L. Haileyesus T. Annest J. (2015). Firearm injuries in the United States. Preventive Medicine, 79, 5–14. 26116133 10.1016/j.ypmed.2015.06.002 PMC4700838

[bibr27-15579883241255830] Freud S. (1922). Introductory lectures on psycho-analysis: A course of twenty-eight lectures delivered at the University of Vienna. Unwin Brothers.

[bibr28-15579883241255830] Gani F. Sakran J. Canner J. (2017). Emergency department visits for firearm-related injuries in the United States, 2006–14. Health Affairs, 36, 1729–1738. 28971917 10.1377/hlthaff.2017.0625

[bibr29-15579883241255830] Geniole S. Carré J. (2018). Human social neuroendocrinology: Review of the rapid effects of testosterone. Hormones and Behavior, 104, 192–205. 29885343 10.1016/j.yhbeh.2018.06.001

[bibr30-15579883241255830] Goss K. (2017). The socialization of conflict and its limits: Gender and gun politics in America. Social Science Quarterly, 98, 455–470.

[bibr31-15579883241255830] Gramlich J. (2019). What the data says about gun deaths in the U.S. Pew Research Center. https://www.pewresearch.org/fact-tank/2019/08/16/what-the-data-says-about-gun-deaths-in-the-u-s/

[bibr32-15579883241255830] Hall C. (1953). A cognitive theory of dream symbols. The Journal of General Psychology, 48, 169–186.

[bibr33-15579883241255830] Hauser W. Kleck G. (2013). Guns and fear: A one-way street? Crime & Delinquency, 59, 271–291.

[bibr34-15579883241255830] Hepburn L. Matthew Miller M. Azrael D. Hemenway D. (2007). The U.S. gun stock: Results from the 2004 National Firearms Survey. Injury Prevention, 13, 15–19. 17296683 10.1136/ip.2006.013607 PMC2610545

[bibr35-15579883241255830] Hill T. D. Dowd-Arrow B. Burdette A. Hale L. (2020). Gun ownership and sleep disturbance. Preventive Medicine, 132, 105996. 31987978 10.1016/j.ypmed.2020.105996

[bibr36-15579883241255830] Hill T. D. Dowd-Arrow B. Burdette A. Warner T. (2020). Gun ownership and life satisfaction in the United States. Social Science Quarterly, 101, 2121–2136.

[bibr37-15579883241255830] Hill T. D. Dowd-Arrow B. Davis A. Burdette A. (2020). Happiness is a warm gun? Gun ownership and happiness in the United States (1973–2018). SSM-population Health, 10, 100536. 31956693 10.1016/j.ssmph.2020.100536 PMC6957840

[bibr38-15579883241255830] Hill T. D. Dowd-Arrow B. Ellison C. Garcia-Alexander G. Bartkowski J. Burdette A. (2021). Sexual dysfunction and gun ownership in America: When hard data meet a limp theory. American Journal of Men’s Health, 15, 15579883211044342. 10.1177/15579883211044342 PMC8447103 34521291

[bibr39-15579883241255830] Hill T. D. Wen M. Ellison C. G. Wu G. Dowd-Arrow B. Su D. (2021). Modeling recent gun purchases: A social epidemiology of the pandemic arms race. Preventive Medicine Reports, 24, 101634. 34976686 10.1016/j.pmedr.2021.101634 PMC8684007

[bibr40-15579883241255830] Holden W. (2012, December). Colorado columnist: Assault rifle owners have “tiny penises.” CoolCleveland. https://kdvr.com/news/colorado-columnist-assault-rifle-owners-have-tiny-penises/

[bibr41-15579883241255830] Kahan D. Braman D. (2003). More statistics, less persuasion: A cultural theory of gun-risk perceptions. University of Pennsylvania Law Review, 151, 1291–1327.

[bibr42-15579883241255830] Kalesan B. Villarreal M. Keyes K. Galea S. (2016). Gun ownership and social gun culture. Injury Prevention, 22, 216–220. 26124073 10.1136/injuryprev-2015-041586 PMC4809774

[bibr43-15579883241255830] Kanabar R. Mazur A. Plum A. Schmied J. (2022). Correlates of testosterone change as men age. The Aging Male, 25, 29–40. 34983291 10.1080/13685538.2021.2023493

[bibr44-15579883241255830] Kanyongo G. Y. Brook G. P. Kyei-Blankson L. Gocmen G. (2007). Reliability and statistical power: How measurement fallibility affects power and required sample sizes for several parametric and nonparametric statistics. Journal of Modern Applied Statistical Methods, 6, 81–90.

[bibr45-15579883241255830] Kelley S. M. (1995). Giggles and Guns: The phallic myth in unforgiven. Journal of Film and Video, 47, 98–105.

[bibr46-15579883241255830] Kish L. (1965). Survey sampling. John Wiley.

[bibr47-15579883241255830] Kleck G. (1997). Targeting guns: Firearms and their control. Aldine.

[bibr48-15579883241255830] Lee P. (1996). Survey report: Concept of penis size. Journal of Sex & Marital Therapy, 22, 131–135. 8743625 10.1080/00926239608404917

[bibr49-15579883241255830] Lever J. Frederick D. Peplau L. (2006). Does size matter? Men’s and women’s views on penis size across the lifespan. Psychology of Men & Masculinity, 7, 129.

[bibr50-15579883241255830] Miller M. Azrael D. Hemenway D. (2002). Rates of household firearm ownership and homicide across US regions and states, 1988–1997. American Journal of Public Health, 92, 1988–1993. 12453821 10.2105/ajph.92.12.1988 PMC1447364

[bibr51-15579883241255830] Miller M. Hemenway D. Azrael D. (2007). State-level homicide victimization rates in the US in relation to survey measures of household firearm ownership, 2001-2003. Social Science & Medicine, 64, 656–664. 17070975 10.1016/j.socscimed.2006.09.024

[bibr52-15579883241255830] Moffic H. (2013, March 5). The psychology of guns: 12 steps toward more safety. Psychiatric Times. https://www.psychiatrictimes.com/view/psychology-guns-12-steps-toward-more-safety

[bibr53-15579883241255830] Nathenson R. (2020). Finding your inner gun: A Jungian perspective on mass shootings and American gun culture. Psychological Perspectives, 63, 204–215.

[bibr54-15579883241255830] Neville-Shepard R. Kelly C. (2020). Whipping it out: Guns, campaign advertising, and the White masculine spectacle. Critical Studies in Media Communication, 37, 466–479.

[bibr55-15579883241255830] Oates J. Sharp G. (2017). Nonsurgical medical penile girth augmentation: Experience-based recommendations. Aesthetic Surgery Journal, 37, 1032–1038. 28498879 10.1093/asj/sjx068

[bibr56-15579883241255830] Parker K. Horowitz J. Igielnik R. Oliphant J. Brown A. (2017, June 22). America’s complex relationship with guns: An in-depth look at attitudes and experiences of U.S. adults. Pew Research Center. https://www.pewresearch.org/social-trends/2017/06/22/americas-complex-relationship-with-guns/

[bibr57-15579883241255830] Pew Research Center. (2021). Methodology: The American Trends Panel survey methodology. https://www.Pewresearch.org/politics/2021/05/17/scope-of-government-methodology/

[bibr58-15579883241255830] Pfaffendorf J. Davis A. Kinney A. (2021). Masculinity, ritual, and racialized status threat: Examining mass shooter manifestos using structural topic models. Sociological Inquiry, 91, 287–312.

[bibr59-15579883241255830] Potts A. (2000). The “essence of the hard on”: Hegemonic masculinity and the cultural construction of “erectile dysfunction.” Men and Masculinities, 3, 85–103.

[bibr60-15579883241255830] Roy J. (2013, December 3). Which state is the biggest? On this list, Texas doesn’t even make the top 40. Time. https://newsfeed.time.com/2013/12/03/which-state-is-the-biggest/

[bibr61-15579883241255830] Sasson-Levy O. (2003). Feminism and military gender practices: Israeli women soldiers in ‘masculine’ roles. Sociological Inquiry, 73, 440-465.

[bibr62-15579883241255830] Sharp G. Fernando A. N. Kyron M. Oates J. McEvoy P. (2022). Motivations and psychological characteristics of men seeking penile girth augmentation. Aesthetic Surgery Journal, 42, 1305–1315. 35511228 10.1093/asj/sjac112 PMC9558456

[bibr63-15579883241255830] Smith T. Laken F. Son J. (2019). Trends in gun ownership in the United States, 1972-2018. National Opinion Research Center.

[bibr64-15579883241255830] Smith T. Smith R. (1995). Changes in firearms ownership among women, 1980-1994. Journal of Criminal Law and Criminology, 86, 133–149.

[bibr65-15579883241255830] Spitzer S. Staudenmayer K. Tennakoon L. Spain D. Weiser W. (2017). Costs and financial burden of initial hospitalizations for firearm injuries in the United States, 2006–2014. American Journal of Public Health, 107, 770–774. 28323465 10.2105/AJPH.2017.303684 PMC5388949

[bibr66-15579883241255830] Stroud A. (2012). Good guys with guns: Hegemonic masculinity and concealed handguns. Gender & Society, 26, 216–238.

[bibr67-15579883241255830] Timbs L. (2023). The politics of the penis: Post-Apartheid Zulu nationalism and martial masculinity, ca 1999 to 2018. African Studies, 82, 99–121.

[bibr68-15579883241255830] Todd K. P. Thornburgh S. Pitter R. Gamarel K. E. Peitzmeier S. (2022). Masculine identity development and health behaviors in transmasculine individuals: A theory of gender and health. SSM-Qualitative Research in Health, 2, 100186.

[bibr69-15579883241255830] Tonso W. (1982). Gun and society: The social and existential roots of the American attachment to firearms. University Press of America.

[bibr70-15579883241255830] Urbatsch R. (2019). Gun-shy: Refusal to answer questions about firearm ownership. The Social Science Journal, 56, 189–195.

[bibr71-15579883241255830] Van Kesteren J . (2014). Revisiting the gun ownership and violence link: A multilevel analysis of victimization survey data. British Journal of Criminology, 54, 53–72.

[bibr72-15579883241255830] Veale D. Eshkevari E. Read J. Miles S. Troglia A. Phillips R. . . .Muir G. (2014). Beliefs about penis size: Validation of a scale for men ashamed about their penis size. The Journal of Sexual Medicine, 11, 84–92. 24118940 10.1111/jsm.12294

[bibr73-15579883241255830] Veale D. Miles S. Bramley S. Muir G. Hodsoll J. (2015). Am I normal? A systematic review and construction of nomograms for flaccid and erect penis length and circumference in up to 15 521 men. BJU International, 115, 978–986. 25487360 10.1111/bju.13010

[bibr74-15579883241255830] Veale D. Miles S. Read J. Troglia A. Carmona L. Fiorito C. . . .Muir G. (2015). Phenomenology of men with body dysmorphic disorder concerning penis size compared to men anxious about their penis size and to men without concerns: A cohort study. Body Image, 13, 53–61. 25675864 10.1016/j.bodyim.2014.09.008

[bibr75-15579883241255830] Willer R. Rogalin C. L. Conlon B. Wojnowicz M. T. (2013). Overdoing gender: A test of the masculine overcompensation thesis. American Journal of Sociology, 118, 980–1022.

[bibr76-15579883241255830] Wylie K. Eardley I. (2007). Penile size and the “small penis syndrome.” BJU International, 99, 1449–1455. 17355371 10.1111/j.1464-410X.2007.06806.x

[bibr77-15579883241255830] Zimmerman D. (2017, April 13). Study confirms gun owners have smaller penises. The Truth About Guns. https://www.thetruthaboutguns.com/study-confirms-gun-owners-smaller-penises-content-contest/

[bibr78-15579883241255830] Zitzmann M. (2020). Testosterone, mood, behaviour and quality of life. Andrology, 8, 1598–1605. 32657051 10.1111/andr.12867

